# Supplementary material for: Downstream Effects of Mutations in SOD1 and TARDBP Converge on Gene Expression Impairment in Patient-Derived Motor Neurons
Source: Int J Mol Sci. 2022 Aug 25;23(17):9652. doi: 10.3390/ijms23179652 (PMC9456253; doi:10.3390/ijms23179652)
Supplement: Supplementary file 1 [file ijms-23-09652-s001.zip › ijms-1842828-supplementary.pdf]

Supplementary material to manuscript entitled

"Downstream Effects of Mutations in *SOD1* and *TARDBP* Converge on Gene Expression Impairment in Patient-Derived Motor Neurons"

Authors

Banaja P. Dash, Axel Freischmidt, Jochen H. Weishaupt and Andreas Hermann

This file includes:

Supplementary figures S1 to S2 with legends

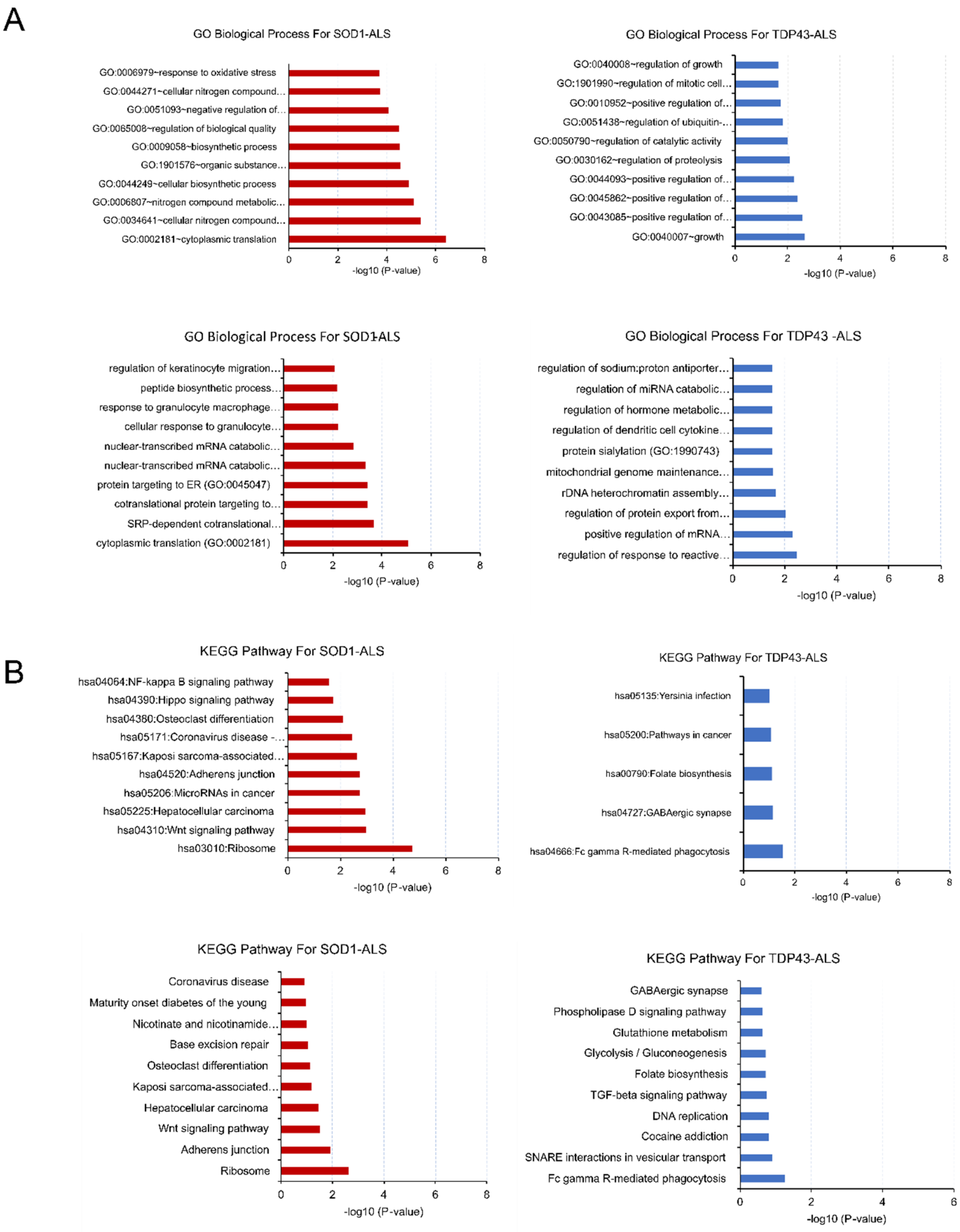

Figure S1. The functional enrichment analysis of all DEGs (*P*-value ≤ 0.05, FC ≥ 0.05) across SOD1- and TDP43-ALS datasets by DAVID and EnrichR. (A) Gene Ontology (GO, Biological Process) analysis was performed by DAVID (upper part) and EnrichR (lower part). (B) KEGG pathways that were significantly enriched in DEGs identified using DAVID (upper) and EnrichR (lower). The statistical significance of the enrichment (-log10(*P*-value)) was reported for each category. KEGG: Kyto Encyclopedia of Genes and Genomes.

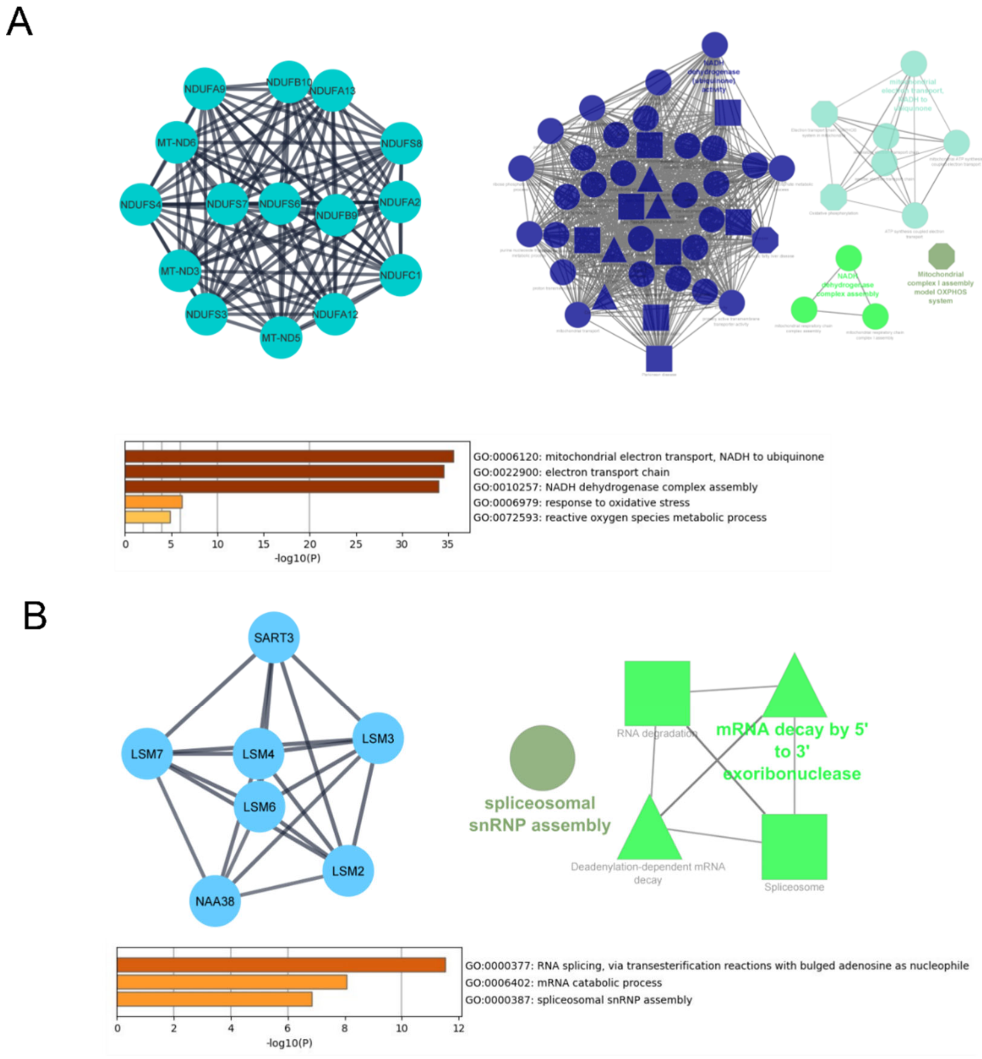

Figure S2. Module analysis of all the DEGs in SOD1- and TDP43-ALS. (A) In SOD1-ALS datasets, the module 2 identified from the whole PPI network (upper left) and comprehensive enrichment analysis of the group network (GO/KEGG terms) was performed using the ClueGo/CluePedia plugin in Cytoscape (upper right) and GO (Biological process) by Metascape (lower part). (B) The module 2 identified from the whole PPI network of TDP43-ALS datasets (upper left). Comprehensive enrichment analysis (GO/Pathway terms) of the group network was generated using the ClueGo/CluePedia plugin in Cytoscape (upper right) and categories (Biological Process) in lower part was performed by Metascape. Lines representing an interacting relationship between nodes. PPI: protein-protein interaction. Statistical significance of *p*-value ≤ 0.05 was applied in the network.

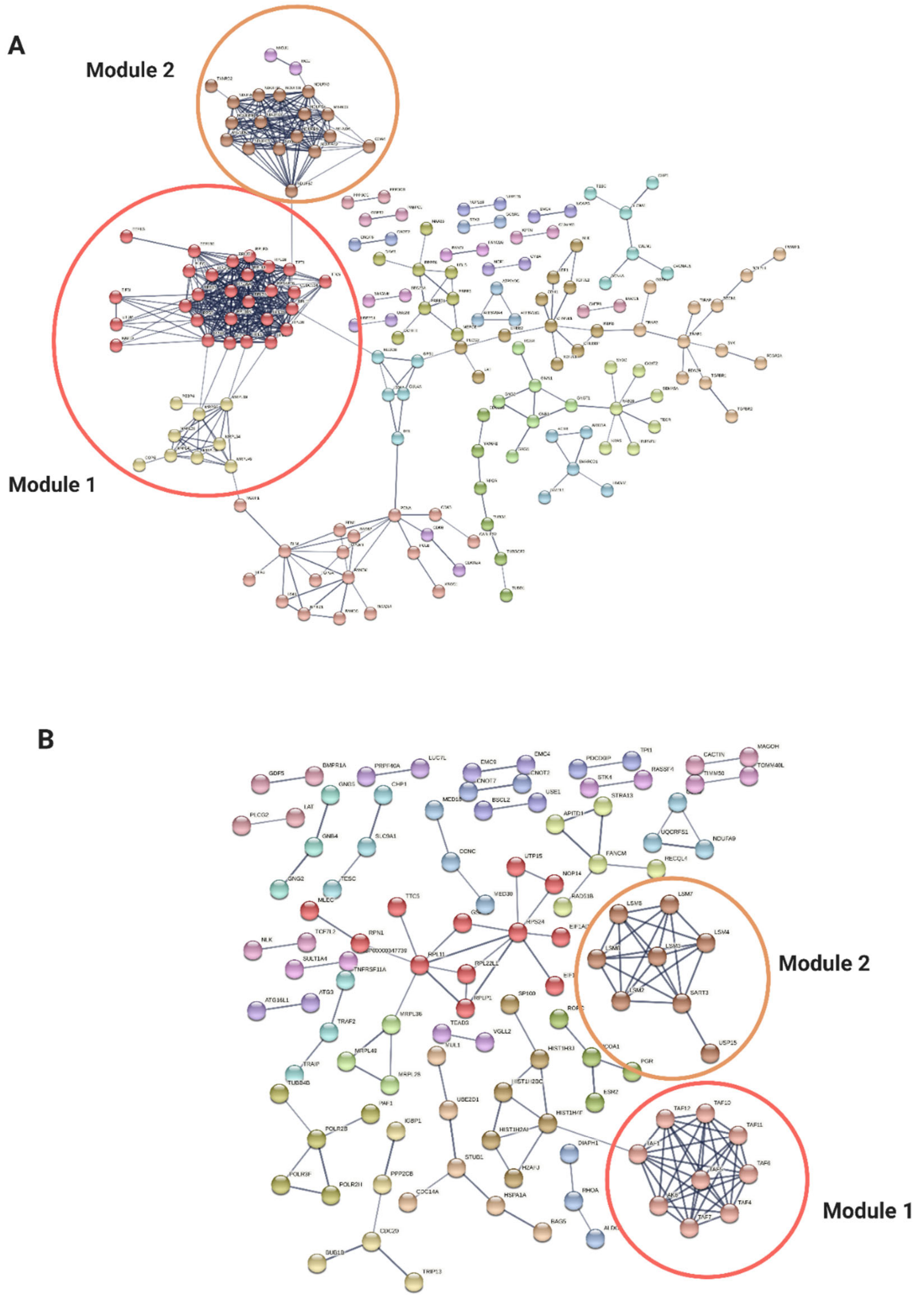

Figure S3. (A) PPI network of SOD1- and (B) TDP43-ALS DEGs, in which thicker lines indicates stronger data support. The nodes indicate the DEGs and the edges indicate the interaction (experimental evidence only) between two proteins. The STRING database was used to establish

functional associations among the known and predicted proteins using annotated DEGs as query for SOD1- and TDP43-ALS interaction networks, with high confidence score of > 0.7 and a maximum number of interactions to top 20. The functional modules with enriched proteins are highlighted.

References:

1. Szklarczyk, D.; Gable, A.L.; Lyon, D.; Junge, A.; Wyder, S.; Huerta-Cepas, J.; Simonovic, M.; Doncheva, N.T.; Morris, J.H.; Bork, P.; et al. STRING v11: Protein-protein association networks with increased coverage, supporting functional discovery in genome-wide experimental datasets. *Nucleic Acids Res.* **2019**, *47*, D607–D613, doi:10.1093/nar/gky1131.
2. Bindea, G.; Mlecnik, B.; Hackl, H.; Charoentong, P.; Tosolini, M.; Kirilovsky, A.; Fridman, W.H.; Pagès, F.; Trajanoski, Z.; Galon, J. ClueGO: A Cytoscape plug-in to decipher functionally grouped gene ontology and pathway annotation networks. *Bioinformatics* **2009**, doi:10.1093/bioinformatics/btp101.
3. Bindea, G.; Galon, J.; Mlecnik, B. CluePedia Cytoscape plugin: Pathway insights using integrated experimental and in silico data. *Bioinformatics* **2013**, doi:10.1093/bioinformatics/btt019.
4. Bader, G.D.; Hogue, C.W.V. An automated method for finding molecular complexes in large protein interaction networks. *BMC Bioinformatics* **2003**, doi:10.1186/1471-2105-4-2.
5. Zhou, Y.; Zhou, B.; Pache, L.; Chang, M.; Khodabakhshi, A.H.; Tanaseichuk, O.; Benner, C.; Chanda, S.K. Metascape provides a biologist-oriented resource for the analysis of systems-level datasets. *Nat. Commun.* **2019**, doi:10.1038/s41467-019-09234-6.
